# *Bordetella pertussis* in sporadic and outbreak settings in Alberta, Canada, July 2004 – December 2012

**DOI:** 10.1186/1471-2334-14-48

**Published:** 2014-01-30

**Authors:** Sumana Fathima, Christina Ferrato, Bonita E Lee, Kimberley Simmonds, Lin Yan, Shamir N Mukhi, Vincent Li, Linda Chui, Steven J Drews

**Affiliations:** 1 Provincial Laboratory for Public Health (ProvLab) Calgary Site, Calgary, AB, Canada; 2 University of Alberta, Edmonton, AB, Canada; 3 Alberta Health, Edmonton, AB, Canada; 4 University of Calgary, Department of Community Health Sciences, Calgary, AB, Canada; 5 Provincial Laboratory for Public Health (ProvLab) Edmonton Site, Edmonton, AB, Canada; 6 Canadian Network for Public Health Intelligence, Public Health Agency of Canada, Winnipeg, MB, Canada; 7 Department of Microbiology, Immunology and Infectious Diseases, University of Calgary, Calgary, AB, Canada

**Keywords:** Pertussis, Outbreaks, Epidemiology

## Abstract

**Background:**

ProvLab Alberta provides all laboratory testing for *Bordetella pertussis* including sporadic cases and outbreak investigations through collaborations with provincial public health partners*.* We describe *B. pertussis* activity in Alberta from July 2004 to December 2012.

**Methods:**

Laboratory testing for pertussis was analyzed using interpreted laboratory data that was generated by DIAL, a secure web-based platform. Duplicate specimens from the same individual ≤90 days were excluded to generate a case-based dataset. Immunization status of confirmed pertussis cases from the provincial immunization repository was reviewed.

**Results:**

Overall, 7.1% of suspected pertussis cases tested positive with a higher positivity rate in outbreak as compared to sporadic setting. Annual variations in sporadic pertussis cases were observed across the province with higher positivity rates in 2005, 2008, 2009 and 2012. A significantly higher positivity rate was observed in a northern region of Alberta. While the positivity rate in sporadic setting was highest in adolescents aged 10 to <15 years old (14.8%), population-based disease burden was highest in young children <5 years old. Of the 81.6% (n = 1,348) pertussis cases with immunization records, 48.3% were up-to-date with immunization. The pertussis cases that were up-to-date with their immunization were older (median age 12.9 years) as compared to those with incomplete (median age 9.7 years) or no pertussis immunization (median age 3.8 years).

**Conclusions:**

Cyclic pattern of annual pertussis activity with geographic variation was observed in Alberta with no obvious case finding effect from outbreak investigations. The high positivity rates in adolescents suggested an underestimation of disease burden in this age group.

## Background

*Bordetella pertussis* is a highly contagious bacterial pathogen that only infects humans and causes respiratory infections that has a relatively predictable clinical course including a catarrhal, paroxysmal and convalescent phase [1-4]. Clinical severity of pertussis is highly variable depending on the age at diagnosis, immunization status and type of vaccine. Morbidities and mortality of pertussis infections are highest in infants <3 months of age and hospitalization rates in infants <1 year remained high in most countries despite vaccine programs [2,4]. Analysis of data from the Italian Trial on Acellular Pertussis Vaccines with three distinct study periods showed that the proportion of children with spasmodic cough, apnea, cyanosis, and vomiting as well as the duration of cough was lower in vaccinated children compared to those without vaccine and that pertussis was milder after 3 years of age. Using a scale to rank key clinical symptoms of pertussis, Preziosi *et al*. showed an overall 48% vaccine efficacy in reducing disease severity among children with a higher efficacy with whole-cell (67%) as compared to acellular (32%) vaccine [5]. Pertussis infections usually result in milder and atypical illness in adolescents and adults and a high index of suspicion is required for making the diagnosis [4,6]. Adults have been identified as an important source of infection of pertussis for neonates and young infants who are at highest risk for severe morbidity and mortality.

Alberta is a province in western Canada, with a population of 3.8 million people as of 2012 with an almost equal number of males and females. Eighteen percent of the population belong to the age group 0–14 years, 70% in the ages of 15–64 years and 11% are over 65 years old [6]. Whole cell vaccine for pertussis was first introduced in Alberta in 1939 and was replaced by acellular pertussis vaccine in 1997 because of a lower side effect profile [7]. Routine pertussis immunization in Alberta includes a combination vaccine that protects against diphtheria, tetanus, acellular pertussis, polio, and *Haemophilus influenzae* type b which is given at 2, 4, 6 and 18 months and a booster dose of diphtheria, tetanus, acellular pertussis and inactivated polio at 4–6 years of age. Before 2004, a booster dose of only tetanus and diphtheria was given in grade 9. As of September 2004, Adacel™, a combined diphtheria, tetanus, and acellular pertussis vaccine for adolescents replaced the booster dose of Td in the grade 9 school immunization program to provide additional protection against pertussis for children in this age group. The infant formulation of the pertussis vaccine, with tetanus and diphtheria +/- inactivated polio +/- *Haemophilus influenza* b vaccine, contains higher concentrations of *B. pertussis* antigens and is used in children from 2 months until 7 years of age. The adolescent/adult formulation contains lower concentrations of pertussis antigens and diphtheria toxoid and is given to persons ≥7 years [8].

Pertussis surveillance in Canada is maintained by two national systems: Canadian Notifiable Disease Surveillance System and The Immunization Monitoring Program ACTive (IMPACT). There has been a decrease of the incidence of pertussis in Canada since the introduction of pertussis vaccines in the 1940’s. A 4-year periodicity for epidemic pertussis has been observed in the post-vaccine era in Canada [9] and an increase of pertussis activity was noted in Alberta in 2012. Pertussis is a notifiable disease in Alberta and under the Public Health Act all cases that meet the case definition are to be reported to the office of the Chief Medical Officer of Health. Alberta uses the Public Health Agency of Canada case definition of pertussis which includes i) laboratory confirmation of *B. pertussis* by positive polymerase chain reaction (PCR) or isolation of this pathogen from a clinical specimen or ii) a person who is epidemiologically linked to a laboratory confirmed case having one or more of the following symptoms: paroxysmal cough of any duration, cough ending in vomiting or with apnea, cough with inspiratory “whoop” [10]. In this study, we examined the laboratory testing for pertussis and the epidemiology of all laboratory confirmed cases of pertussis in Alberta from July 1, 2004 to December 31, 2012.

## Methods

### Laboratory testing for pertussis

The Provincial Laboratory for Public Health (ProvLab) operates as a single laboratory with two sites, Edmonton and Calgary, in the province of Alberta. ProvLab undertakes diagnostic testing for all cases of *B. pertussis* in Alberta and surrounding Northern Territories. ProvLab also provides laboratory testing for all outbreak investigations through collaborations with provincial public health partners. Prior to June 2004, direct fluorescent antibody test and/or culture for *B. pertussis* was used to test slides and/or nasopharyngeal swabs submitted to ProvLab for pertussis testing. Since June 2004, ProvLab has used a real-time PCR assay for the detection of the *IS*481 element within *B. pertussis* from respiratory specimens [11]. Specimens are reported as positive for *B. pertussis* if the amplification curve is of good quality and the crossing point is ≤ 35 cycles. The melting curves of specimens that have a good quality amplification curve and a crossing point >35 cycles are reviewed by technical supervisor and microbiologist. A specimen that cannot be interpreted as either positive or negative is reported as indeterminate. All specimens tested positive by PCR are cultured for *B. pertussis* on Regan-Lowe plates and colonies with morphology and gram stain compatible with *Bordetella* species are tested using Accu-MAb™ Plus Bordetella pertussis/parapertussis DFA (Delta Biotech Inc., British Columbia, Canada) for identification. Specimens associated with a suspected *B. pertussis* outbreak are assigned a specific Exposure Investigation (EI) number for each outbreak.

### Web-based platform for laboratory surveillance

Data Integration for Alberta Laboratories (DIAL) is a secure web-based platform that is being used in ProvLab to extract, interpret, collate and analyze testing data [12]. DIAL extracts raw specimen data from ProvLab’s Laboratory Information System and with its built-in automated interpretation engine, provides clinical interpretive results for specific pathogens. In the case of *B. pertussis*, DIAL automatically assigns a final classification to each specimen as PCR-positive, negative or indeterminate for pertussis and indicates whether the *B. pertussis* isolate was cultured and archived. ProvLab provides laboratory investigations for all outbreaks in Alberta and the information related to all the outbreaks is also available on the DIAL platform, including the location, setting and geographic information of these outbreaks, which can be easily accessed and analyzed.

### Case-based data for pertussis

Line list data for all specimens received at ProvLab for *B. pertussis* testing from July 1, 2004 to December 31, 2012 were extracted from DIAL with patient demographics and submitter information. DIAL assigns geographic designation for specimens in the order of availability of patient’s city of residence, submitting physician location, and submitting location (hospital or clinic) in the Laboratory Information System. Only specimens from Alberta were included in this study. An early study by van der Zee *et al.* showed that 8-25% of the specimens still tested positive by PCR at >30 days of illness [13]. Specimens collected ≤ 90 days from the same individual were excluded to create a stringent criterion to eliminate duplicate specimens and create a case-based dataset. Immunization records of confirmed pertussis cases were linked to the provincial immunization repository and the Communicable Disease Reporting System to determine the immunization status of the cases [14].

### Sporadic versus outbreak cases

All cases with no associated Exposure Investigation (EI) number related to outbreak investigations were classified as sporadic cases. For cases that were associated with an EI number, we used DIAL to extract the location and setting of the outbreak and classified each EI as either community-based outbreak (COB) or facility-based outbreak (FOB). COBs were defined as outbreaks in geographically-defined communities with higher than normal pertussis activity. FOBs were defined as outbreaks in facilities including schools, daycares, long-term care, assisted living facilities, hospitals, households, and other congregated settings. The age of the cases tested in school outbreaks was reviewed as some of the school outbreaks were extended to community-based investigations. School (grade 1–12) outbreaks where >25% of the cases tested were younger than 2 years or older than 20 years were reclassified as COBs.

### Data analysis

Excluding specimens tested as indeterminate for pertussis, the positivity rate of pertussis in sporadic settings, COBs and FOBs were compared using the chi-squared test. Differences in positivity rate by annual distribution, gender, and age groups in each setting were analyzed using binary logistic regressions with Bonferroni correction for multiple comparisons performed for each variable because of the three settings. Difference in geographic distribution of positivity rate was performed only for sporadic cases because of cases coming from multiple regions for outbreaks designated in a single region as related to travel. For annual comparison, data from January to June 2004 was excluded as there were only 6 months of data for 2004. Cases with unknown gender or age were excluded from the respective comparison. The age of the confirmed pertussis cases in sporadic and the two outbreak settings was compared using the Kruskal-Wallis test. All statistical analyses were performed using IBM SPSS Statistics Version 20.0.0. Crude population rate by age group from 2005 to 2012 for all laboratory confirmed pertussis cases was calculated using population data from Statistics Canada [6].

## Results

A total of 26,487 specimens were received at ProvLab from July 1, 2004 to December 31, 2012 for *B. pertussis* testing and 1,404 specimens were from out of province and excluded from this study. Of the 25,083 specimens from Alberta, 1,377 (5.5%) were also excluded because pertussis testing was not performed due to reasons that include inappropriate specimen types or transport media, incomplete patient or submitter information, and leakage during specimen transport. Of the remaining specimens from Alberta, 454 patients had one or more specimen submitted ≤90 days resulting in 500 (2.1%) specimens being excluded as duplicates: 416 individuals had one duplicate specimen, 33 had two, four had three and one individual had six duplicate specimens. The median time between duplicate specimens was 21 days (interquartile range: 3–48 days). A total of 23,206 suspected pertussis cases were included in the study with 21,784 (93.9%) cases from patients with only one specimen and 1,422 (6.1%) cases from 681 patients with >1 specimen per patient. The median time period between specimens included from an individual was 432 days (interquartile range: 216–920 days).

During the study period, one or more specimens were submitted for pertussis testing for 45 outbreak investigations: 16 investigations were initiated in geographically defined communities, nine in schools, and the remaining outbreaks in a variety of settings including long-term care or assisted living facilities (n = 9), households or community gatherings (n = 6), hospitals (n = 2), daycare (n = 1), and other settings (n = 2). Five of the nine school outbreaks were extended to community-based investigations with a total of 283 of 887 (31.9%) suspected cases in these outbreaks (range: 26.8-33.9%) being younger than 2 years or older than 20 years and were reclassified as COBs.

**Figure 1:**
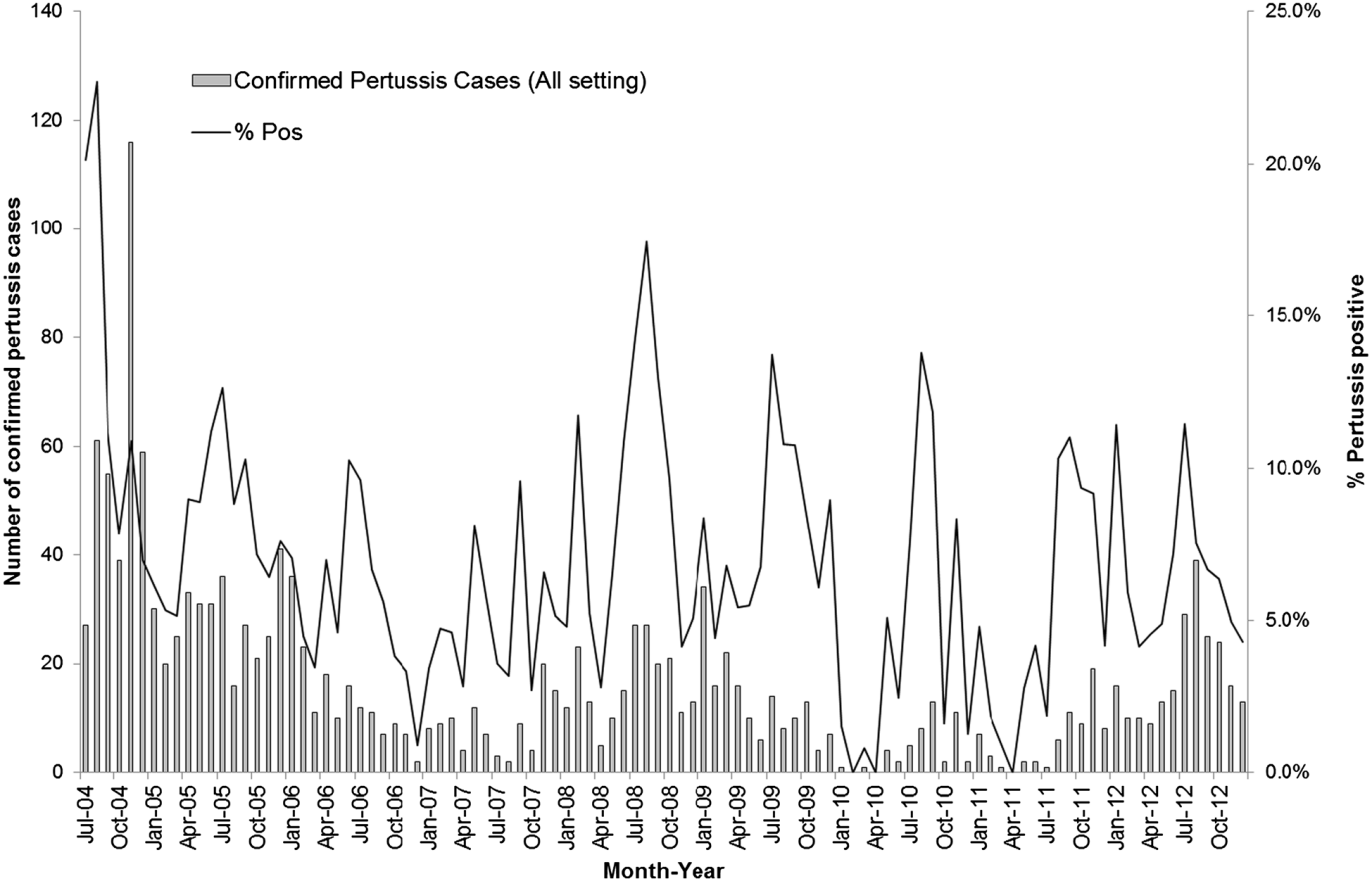
**Monthly distribution of confirmed *B. pertussis *** **cases and test positivity rate in Alberta (July 2004 – December 2012).**

**Figure 2:**
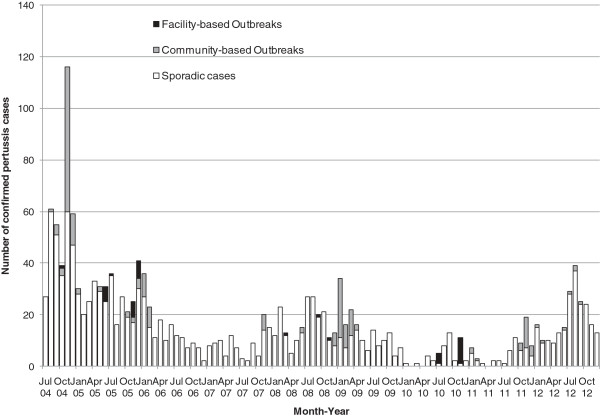
**Monthly distribution of confirmed *B. pertussis *** **cases by the type of setting: sporadic, community-based and facility-based outbreaks in Alberta (July 2004 – December 2012).**

After removing duplicate specimens, a total of 21,484 suspected sporadic pertussis cases, 1,598 suspected cases from 21 COBs, and 124 suspected cases from 24 FOBs were investigated for pertussis. Overall, 7.1% (1,652/23,206) cases tested positive for *B. pertussis* and 8 sporadic cases tested as indeterminate. There was significant difference in the positivity rate among the different settings, with 6.6% for sporadic cases, 12.0% for COBs and 30.6% for FOBs respectively (p < 0.001, chi-squared test) (Table 1). The monthly distribution of overall test positivity rate for pertussis is presented in Figure 1 and the pertussis cases by the type of setting in Figure 2. The months of July, August and September were most commonly identified as the months with the top three positive rates for pertussis from 2005 to 2012. The annual distribution of suspected and confirmed sporadic pertussis cases and cases associated with COBs and FOBs is summarized in Table 1. There was no COB in 2010 and no FOB in 2011. July to December 2004 had the highest number of outbreaks with six COBs and five FOBs and this time period also had the highest positivity rate for pertussis (Table 1). Excluding the six months of data from 2004 and using 2005 as the reference year, significantly lower positive rates were observed for sporadic pertussis in 2006 (5.1%), 2007 (4.8%), 2010 (3.0%) and 2011 (4.1%) as compared to 2005 (7.3%) (p < 0.01, binary logistic regression with Bonferroni correction). The year 2008 had significantly different positivity rates when compared to 2005 for COBs and FOBs (p < 0.05 and p < 0.01, respectively, binary logistic regression with Bonferroni correction); the rate was high for COBs in 2008 (50%) and low for FOBs in 2008 (9.4%).

**Table 1:** **Annual distribution of *B. pertussis *** **in sporadic, community-based and facility-based outbreak settings**

	**Sporadic cases**		**Community-based outbreak**			**Facility-based outbreak**		
	**Suspected**	**Confirmed (%)**	**No. of outbreaks**	**Suspected**	**Confirmed (%)**	**No. of outbreaks**	**Suspected**	**Confirmed (%)**
Complete period	21,484	1,423 (6.6)^1^	21	1,598	191 (12.0)^1^	24	124	38 (30.6)^1^
2004*	2,611	280 (10.7)	6	678	76 (11.2)	5	14	1 (7.1)
2005^2,3,4^	4,175	304 (7.3)	3	79	12 (15.2)	4	42	20 (47.6)
2006	2,841	145 (5.1)^2^	2	220	17 (7.7)	1	1	0 (0.0)
2007	2,023	97 (4.8)^2^	1	6	6 (100.0)	2	11	0 (0.0)
2008	2,359	187 (7.9)	1	14	7 (50.0)^3^	5	32	3 (9.4)^4^
2009	1,905	116 (6.1)	3	319	44 (13.8)	3	4	0 (0.0)
2010	1,161	35 (3.0)^2^	0	0	NA	3	19	14 (73.7)
2011	1,157	47 (4.1)^2^	4	158	22 (13.9)	0	0	NA
2012	3,252	212 (6.5)	1	124	7 (5.6)	1	1	0 (0.0)

*Only 6 months of data included for 2004 (July to December).

^1^p < 0.001, Chi-squared test excluding eight sporadic cases with indeterminate PCR results.

^2^p < 0.01, binary logistic regression with Bonferroni correction using 2005 as the reference year excluding 2004 (6 months only).

^3^p < 0.05, binary logistic regression with Bonferroni correction using 2005 as the reference year excluding 2004 (6 months only) and 2010 (no community-based outbreak).

^4^p < 0.05, binary logistic regression with Bonferroni correction using 2005 as the reference year excluding 2004 (6 months only) and 2011 (no facility-based outbreak).

Gender and age distribution for all three settings is shown in Table 2. There was no significant difference in terms of gender distribution for the different settings. Excluding cases with unknown age and using the ≤8 weeks age group as the reference group, significantly higher positive rates were identified in the 10 to <15 years (14.8%) and 15 to <20 years (9.4%) age categories in the sporadic setting. No significant difference was found in the positivity rate among age groups in COBs and FOBs. There was no significant difference in the median age of confirmed pertussis cases in the sporadic setting, COBs and FOBs: 12.0 years (range: 0.05 – 83 years), 11.3 years (range: 0.2 – 51 years), and 11.4 years (range: 0.5 – 38 years), respectively (p > 0.05, Kruskal-Wallis test). The overall positivity rate and crude population rate of all laboratory-confirmed pertussis cases from 2005 to 2012 by age groups is summarized in Table 3. While the positivity rate is highest in the 10 to <15 year age group at 14.8% (Table 1), the crude population rate of laboratory-confirmed pertussis cases is highest in children younger than 5 years (27.5%) (Table 3).

**Table 2:** **Gender and age distribution of *B. pertussis *** **in sporadic, community-based and facility-based outbreak settings**

	**Sporadic cases**		**Community-based outbreaks**		**Facility-based outbreaks**	
Gender ratio	Suspected	Confirmed	Suspected	Confirmed	Suspected	Confirmed
Male: Female*	1:1.1	1:1.2	1:1.3	1:1.0	1:1.4	1:1.1
Age category	Suspected	Confirmed (%)	Suspected	Confirmed (%)	Suspected	Confirmed (%)
≤8 weeks^1^	1,024	55 (5.4)	8	0 (0.0)	0	NA
>8 weeks - <6 months	1,522	107 (7.0)	43	6 (14.0)	3	0 (0.0)
6 months - <18 months	2,414	108 (4.5)	105	15 (14.3)	7	2 (28.6)
18 months – <5 years	3,533	177 (5.0)	246	22 (8.9)	26	10 (38.5)
5 - <10 years	2,739	169 (6.2)	273	41 (15.0)	13	4 (30.8)
10 - <15 years	2,158	319 (14.8)^1^	311	61 (19.6)	22	15 (68.2)
15 - <20 years	1,339	126 (9.4)^1^	224	29 (12.9)	12	2 (16.7)
20 - <40 years	3,412	197 (5.8)	205	8 (3.9)	30	5 (16.7)
40 - <60 years	2,506	137 (5.5)	142	9 (6.3)	3	0 (0.0)
60 years and older	773	26 (3.4)	38	0 (0.0)	8	0 (0.0)
Unknown	64	2 (3.1)	3	0 (0.0)	0	NA

*443 (1.9%) of cases had unknown gender with 422 in sporadic setting and 21 in community-based outbreaks. Seven of the 443 (1.6%) cases tested positive for pertussis with all confirmed cases in the sporadic setting.

^1^p < 0.01, binary logistic regression with Bonferroni correction using ≤8 weeks as the reference group excluding cases with unknown age.

**Table 3:** **Overall positivity rate and crude population rate per 100,000 of confirmed *B. pertussis *** **by age groups in Alberta**

**Age group (number of laboratory confirmed cases)**	**Confirmed (% of suspected cases)**	**Crude population rate of confirmed cases per 100,000**
0 - <5 years (n = 441)*	5.4%	27.5
0- <1 years (n = 209)*	5.6%	62.3
5 years - <10 years (n = 182)	7.1%	10.6
10 years - <15 years (n = 273)	14.1%	15.4
15 years - <20 years (n = 95)	8.2%	4.9
20 years - <40 years (n = 167)	5.4%	1.9
40 years - <60 years (n = 113)	4.9%	1.4
60 years & up (n = 23)	3.2%	0.5

*The 0 - <1 year group is a subset of the 0 - <5 year group.

Immunization records were obtained for 1,348 (81.6%) pertussis cases, out of which 651 (48.3%) were up-to-date for their pertussis immunization, 231 (17.1%) had incomplete series and 466 (34.6%) were not immunized. Excluding the 55 cases who were ≤8 weeks old, thus not yet eligible for pertussis vaccine, there was significant difference in the median age of different groups based on their immunization status at the time of pertussis infection. The median age for those who were up-to-date for immunization was 12.9 years (interquartile range: 8.6-15.3), those with incomplete series were 9.7 years (interquartile range: 3.1-15.3) and those who were not vaccinated were 3.8 years (interquartile range: 0.9-9.0), respectively (P < 0.001, Kruskal-Wallis test).

Alberta was designated into 9 health regions by geographic location in April 2003 and was reorganized into five zones in 2009. For sporadic cases, the positivity rate was significantly higher in Region 9 (12.8%), p < 0.001, binary logistic regression (Table 4). Only the health regions of the cities or the facilities where the COBs and FOBs were initiated are summarized in Table 4. Out of the 21 COBs, 10 had cases that were based in neighbouring regions. Cases involved in FOBs also resided in different health regions from the original outbreak site. Geographic comparison was not performed for COBs and FOBs, however COBs in region 9 had a noticeably high positive rate of 72.2%.

**Table 4:** **Regional distribution of *B. pertussis *** **in Alberta in sporadic, community-based and facility-based outbreak settings**

	**Sporadic cases**		**Community-based outbreaks**			**Facility-based outbreaks**		
Geographic region*	**Suspected**	**Confirmed (%)**	No. of outbreaks*	**Suspected**	**Confirmed (%)**	No. of outbreaks*	**Suspected**	**Confirmed (%)**
Region 1^1^	1,480	96 (6.5)	1	389	44 (11.3)	3	3	0 (0.0)
Region 2	830	37 (4.5)	1	11	0 (0.0)	1	0	NA
Region 3	2,994	171 (5.7)	0	8	1 (12.5)	1	14	12 (85.7)
Region 4	3,027	209 (6.9)	5	849	93 (11.0)	3	19	1 (5.3)
Region 5	863	44 (5.1)	4	90	12 (13.3)	4	22	7 (31.8)
Region 6	6,820	418 (6.1)	1	17	2 (11.8)	5	21	4 (19.0)
Region 7	1,965	129 (6.6)	3	35	2 (5.7)	5	15	6 (40.0)
Region 8	2,172	148 (6.8)	4	181	24 (13.3)	2	17	4 (23.5)
Region 9	1,333	171 (12.8)^1^	2	18	13 (72.2)	0	13	4 (30.8)

*Geographic comparison was not performed for community-based and facility-based outbreaks because outbreaks initiated in a specific region usually involved residents from multiple regions because of travel.

^1^p < 0.001, binary logistic regression using region 1 as the reference group.

## Discussion

Using laboratory testing data we analyzed over eight years of *B. pertussis* activity in the province of Alberta. The DIAL platform was used to extract *B. pertussis* testing data and outbreak information to categorize cases into sporadic or outbreak settings. Reviewing the demographics of cases investigated in individual outbreaks allowed the identification of school outbreaks which had broader impact in the community and analysis of laboratory confirmed pertussis cases in three different settings. The overall disease burden is underestimated as epidemiologically linked cases were not included in the analysis.

Recently, a mixed B. holmesii and B. pertussis outbreak with 30% of holmesii cases was reported in Ohio, United States [15] and some studies identified 3 to 9% of *B. holmesii* in patients of all ages with B. holmesii or B. pertussis using culture or PCR [16-19]. Results from these studies questioned the validity of using PCR assay targeting the IS481 element for B. pertussis surveillance because of the cross-reactivity of the assay for both species. In Alberta, the validation study of the IS481 PCR assay did not identify any B. holmesii from 808 specimens between 2003 and 2004 [20]. In the current study, *Bordetella* species were cultured from ~ 50% of PCR positive specimens and only two specimens tested negative for *B. pertussis* by DFA and both were identified as *B. pertussis* by 16S sequencing (data not shown). While there is still a possibility of B. holmesii in PCR positive and culture negative specimens, the proportion of B. holmesii in Alberta is likely low, which has also been reported in Ontario, Canada (< 2%) and in Finland and Holland (0%) [21]. Readers should be aware that although this cross reactivity has been discussed in many jurisdictions, including Canada, there is no consensus on what molecular targets should be used to identify pertussis. This is due to multiple factors and the authors are working with partners such as the National Molecular Diagnostics User Group in Canada to address these gaps.

In Canada, over 4000 cases of pertussis per year have been reported between 1900 and 2006 [22]. In Alberta, pertussis activity peaked between the months of July and September from 2005 to 2011. An autumn seasonality for pertussis was also reported in other parts of Canada. Between 1990 and 2000 in British Columbia, pertussis were most often found in the months of July and peaked between August and November [23]. A study in Toronto, Ontario, identified most cases of pertussis between August and November [24]. From the international perspective, a large study looking at 16 different European countries examined the epidemiology of pertussis between 1998 and 2002 showed that 28% of pertussis cases occurred from July to September [25].

In the current study, most pertussis cases (86%) in Alberta were identified in the sporadic setting. In 2004, several regions in Alberta experienced outbreaks and the rate of pertussis in the province was 18.8/100,000 cases with a total of 684 reported cases [26]. For the six community-based outbreaks captured from July to December 2004 in this study, three were school outbreaks that had extended to the community and one of those outbreaks contributed to 20% of the 357 outbreak-related cases in the 6-month period (data not shown). Most of the pertussis cases identified through COBs or FOBs in the province were in the winter months, November to March. Thirty-one (68.9%) of the 45 outbreaks with pertussis testing were initiated between November to March, which coincided with the higher incidence of overall respiratory illness and outbreak investigations in the winter.

The overall positivity rate in Alberta using the same PCR assay from June 2004 to December 2005 was 7.1%, which is similar to the 9.4% reported in the Greater Toronto Area from 1993 to 2007 when two PCR assays were utilized overtime [24]. Excluding data from 2004, which represented only six months of data, higher case loads and positivity rates were observed in 2005, 2008, 2009 and 2012 in Alberta. Our results were similar to previous reports with annual peaks and an epidemic cycle of every 2–5 years [9]. It is possible that there were biases for case-finding by increased testing in 2005 and 2012 as there were higher numbers of suspected sporadic cases in those years. On the other hand, no obvious relationship between higher sporadic activity and the number of community-based outbreaks was observed in those years. The reasons behind the changes in pertussis burden over time in Alberta are not known. An ecological cross-sectional study in Australia using archived seroprevalence data showed a higher proportion of low level pertussis antibodies in the sampled population the year before several years of high level pertussis activity suggesting the effect of cyclic waning immunity [27]. A potential research focus can look at emerging genetic polymorphisms in *B. pertussis* clones in Alberta to determine geographical epidemiological trending. Shuel *et al*. looks at *B. pertussis* in Ontario from 1998 to 2006 and has found one predominant clone linked to epidemics in Europe and Australia [28].

Our study did not find a significant difference in gender, which is consistent with previous data from Alberta [26] and Ontario where roughly 50% of specimens submitted were from females [24]. The annual positivity rate for sporadic pertussis was highest for the 10 to <15 years age group (14.8%). This is consistent with earlier Canadian data where rates of 10 to <15 year olds from 2002–2004 were elevated compared to other children [26], and this may be attributed to the lower efficacy of the combined adsorbed diphtheria-tetanus-pertussis whole-cell vaccine used in children in Canada between 1980 and 1997 [29]. Recent resurgence of pertussis with a shift towards higher rate in adolescents and young adults has also been reported in United States and Australia [30,31]. About half of the pertussis cases in our study had completed their immunization series with a median age of 12.9 years. Two studies have shown a progressive increase in the odds for pertussis infection with time passed since the last acellular pertussis dose in children with five doses of vaccines supporting the role of waning immunity in this population [28,32]. Because of the change in diagnostic assay from culture to PCR in July 2004, pertussis burden before and after the adolescent booster dose that was implemented in September 2004 cannot be compared.

While the *B. pertussis* positivity rate was highest in young adolescents, children younger than 5 years remained as the group with highest disease burden at the population level in Alberta. The lower test positivity rate in infants and young children in sporadic setting could be due to over sampling, which suggested that the disease burden in adolescent and young adults were underestimated in Alberta with their high test positivity rate. One extensive case-based study found that household contacts were the cause of 48-55% of *B. pertussis* transmission to the <1 year age group, which support the importance of disease control and prevention in adolescents and adults [33]. The severity of pertussis in young infants was shown in a study in 25 pediatric intensive care units in United Stated where 83% of the 124 critical cases were younger than 3 months old and had a 10% mortality rate [34]. In September 2012, United Kingdom started a pertussis vaccination program for pregnant women in their third trimester in September 2012 as a response to the increase in pertussis in young infants in 2012 [35]. In the United States, a recent study followed a 2009 birth cohort (4,131,019 infants) and found that trimester dTAP vaccination of pregnant mothers significantly reduced infant pertussis cases, hospitalizations, and deaths over postpartum cocooning strategies [36]. Clinical trials are still ongoing in Canada to study the efficacy and safety of this approach.

Over the study period, Region 9 had the highest positivity rate in sporadic cases and COBs. Region 9 is the northernmost health region in Alberta, and is mostly rural with multiple small towns and communities. Our finding is consistent with Alberta’s neighbouring province, Saskatchewan, where a 9-year study showed higher incidence of pertussis disease as well as lower vaccination rates in rural areas as compared to urban areas [37]. While lower vaccination rates have also been observed in rural compared to urban areas in the province (data not shown), other factors such as age distribution, population density, and physicians testing behaviour in the different regions might have contributed to the significantly higher positivity rate in Region 9 as compared to other regions that are also comprised of rural areas.

## Conclusion

To our knowledge, this is the first paper in Canada that looks at sporadic versus outbreak pertussis cases and trending across different settings. Innovative platforms such as DIAL support access to specimen-based data and outbreak information required for the study. A cyclical pattern of annual pertussis activity with geographic variation was observed in Alberta. Patients associated with outbreaks would more likely be tested positive. We did not find an obvious relationship of increased case finding in sporadic setting due to outbreak investigations. The high test positivity in the adolescents and young adults compared to other age groups suggested that disease burden in those populations was underestimated.

## Abbreviations

IMPACT: The immunization monitoring program ACTive; EI: Exposure Investigation; DIAL: Data Integration for Alberta Laboratories; PCR: Polymerase chain reaction; COB: Community-based outbreak; FOB: Facility-based outbreak.

## Competing interest

The authors declare that they have no competing interests.

## Authors’ contributions

SF and CF co-wrote the initial draft of the manuscript and BL performed statistical analyses of epidemiological data and revised the manuscript for important intellectual content. SM, BL, LY, LC and VL created the web-based platform (DIAL) for accessing the pertussis and outbreak data and all contributed to critical revision of the manuscript. KS provided immunization data and contributed to critical revision of the manuscript. SD designed the study and contributed to critical revision of the manuscript. All authors read and approved the final manuscript.

## Pre-publication history

The pre-publication history for this paper can be accessed here:

http://www.biomedcentral.com/1471-2334/14/48/prepub
